# Familiarity Bias and Physiological Responses in Contagious Yawning by Dogs Support Link to Empathy

**DOI:** 10.1371/journal.pone.0071365

**Published:** 2013-08-07

**Authors:** Teresa Romero, Akitsugu Konno, Toshikazu Hasegawa

**Affiliations:** 1 Department of Cognitive and Behavioral Sciences, Graduate School of Arts and Sciences, The University of Tokyo, Tokyo, Japan; 2 Japan Society for the Promotion of Sciences, Tokyo, Japan; Institut Pluridisciplinaire Hubert Curien, France

## Abstract

In humans, the susceptibility to yawn contagion has been theoretically and empirically related to our capacity for empathy. Because of its relevance to evolutionary biology, this phenomenon has been the focus of recent investigations in non-human species. In line with the empathic hypothesis, contagious yawning has been shown to correlate with the level of social attachment in several primate species. Domestic dogs (*Canis familiaris*) have also shown the ability to yawn contagiously. To date, however, the social modulation of dog contagious yawning has received contradictory support and alternative explanations (i.e., yawn as a mild distress response) could explain positive evidence. The present study aims to replicate contagious yawning in dogs and to discriminate between the two possible mediating mechanisms (i.e., empathic *vs*. distress related response). Twenty-five dogs observed familiar (dog’s owner) and unfamiliar human models (experimenter) acting out a yawn or control mouth movements. Concurrent physiological measures (heart rate) were additionally monitored for twenty-one of the subjects. The occurrence of yawn contagion was significantly higher during the yawning condition than during the control mouth movements. Furthermore, the dogs yawned more frequently when watching the familiar model than the unfamiliar one demonstrating that the contagiousness of yawning in dogs correlated with the level of emotional proximity. Moreover, subjects’ heart rate did not differ among conditions suggesting that the phenomenon of contagious yawning in dogs is unrelated to stressful events. Our findings are consistent with the view that contagious yawning is modulated by affective components of the behavior and may indicate that rudimentary forms of empathy could be present in domesticated dogs.

## Introduction

Contagious yawning, or yawning after seeing or hearing another individual yawning, is an intriguing phenomenon, and the underlying mechanisms and functions remain unclear [1]. In humans, contagious yawning affects 45–60% of healthy adults, and it has been demonstrated experimentally by exposing individuals to video sequences showing yawns [2], [3]. Although some authors have suggested that contagious yawning is a response to innate releasing mechanisms [2], [4], more recent hypotheses have focused on its potential role in communication, social interactions, and empathy [1], [5], [6]. Evidence from clinical, psychological, behavioral and neurobiological studies has supported this latter view. In humans, yawning when seeing other people yawn is associated with activations in neural networks responsible for empathy and social skills [7]–[9]. Furthermore, people who performed better on tests of self-recognition, theory of mind and empathy were more susceptible to yawn contagiously [1], [3]. A recent naturalistic study has also demonstrated that the social-emotional bond between individuals, associated with empathy [10] affects the occurrence, frequency, and response latency of yawn contagion in humans [11]. Additionally, the contagious effect of yawning seems to be impaired in subjects suffering from empathy disorders, such as autism [12], [13].

The evidence supporting the link between contagious yawning and empathy is not specific to humans. Chimpanzees (*Pan troglodytes*), bonobos (*Pan paniscus*) and gelada baboons (*Theropithecus gelada*) have been reported to yawn contagiously when they observe a conspecific yawning [14]–[19]. Similarly to humans, in both species the closer the social bond between individuals, the more likely they would yawn when the other yawned [16]–[18]. These findings are consistent with the empathic-based hypothesis of contagious yawning since in both humans and animals empathy is biased toward individuals who are more similar, familiar, or socially close [10], [20].

Outside the primate order contagious yawning has received far less attention. It has been demonstrated or suggested only in one species of birds (budgerigars, *Melopsittacus undulates*; [21]) and in the domestic dog (*Canis familiaris*; [22]–[26]). Intriguingly, the only attempts to test the empathic-based, emotionally connected contagious yawning have been done in dogs with contradicting findings.

The first study investigating contagious yawning in dogs showed that a high proportion of the subjects (72%) yawned after observing a human experimenter acting a yawn [22]. The authors argued that since dogs are unusually skilled at reading human social and communicative signals [27] there is the potential that dogs may also have developed the capacity for empathy towards humans, and thus being able to catch human yawns. Following a similar procedure, Madsen & Persson [26] confirmed that dogs are able to yawn contagiously but failed to demonstrate that the emotional closeness with the model affected the strength of contagion. Another recent study, however, has provided data that support the empathic-based explanation of contagious yawning in dogs using auditory stimuli [25]. Silva et al. [25] explored dogs’ reactions to the sound of a human yawn finding that not only dogs yawned contagiously when they heard a human yawning, but that they yawned more at familiar than unfamiliar yawns, thus following the same familiarity bias as empathy.

On the other hand, the other two studies investigating yawn contagion in dogs have found very limited evidence of the phenomenon itself or of the linkage between yawn contagion and empathy [23], [24]. In these studies, dogs did not yawn more frequently when exposed to yawn stimuli than when exposed to control ones, nor were their responses affected by their social bond with the yawner. Instead, the authors suggested that if dogs yawn contagiously then the contagion might rely on less cognitively stringent grounds than empathy [24]. Although it is likely that the different methodologies used in each study contributed to the discrepancy between results (e.g., the use of video *vs*. live presentation of stimuli; human *vs*. dog models; see [28]), the current evidence do not allow firm conclusions to be drawn as to whether or not dogs are able to yawn contagiously or whether the phenomenon is empathy-related.

An additional problem when examining the current evidence on dog contagious yawning is that none of the previous studies allow alternative hypotheses to be dismissed. For instance, spontaneous yawning has been associated with psychological tension or mild stress in several animal species including dogs [29], [30]. Thus, it could be possible that dogs yawn more frequently during a particular condition simply because the stimuli presented increase their anxiety (e.g., hearing, but not seeing, their owners). A similar interpretation was given to apparent contagious yawning in stump-tail macaques (*Macaca arctoides*) since both yawning and self-scratching, which is considered an indicator of tension in primates [29], increased when the monkeys were exposed to a video of conspecifics yawning [31]. Some attempts have been made to address this issue in dog’s experiments. In some studies the authors visually distinguished “tension” yawns from “natural” yawns according to the yawn intensity, or to the association with behavioral indicators of anxiety [23], [25], [26]. However, none of these studies provided an objective definition of yawn intensity that could be replicated by other researchers, nor did they report quantitative data on behavioral indicators of anxiety that could be compared across conditions. Additionally, in one study an acoustic stethoscope was used to take heart rate measures at three time points throughout the experimental session [24]. However, the use of a stethoscope to measure stress inherently disturbs the animals, thereby affecting their stress levels and making accurate assessment of stress difficult. Thus, no study that has so far reported contagious yawning in dogs could rule out the stress-response hypothesis.

If contagious yawning indeed is related to the capacity for empathy, it could became a powerful tool to explore the root of empathy in animal evolution by studying cross-species contagious yawning. Therefore, there is a need for further experimentation on this issue, especially in non-primate species. The current study explores whether contagious yawning can be observed in the domestic dog. In particular, we tested whether dogs yawn when they see a human yawning and whether this response is similar to contagious yawning observed in humans and other primates or is due to tension or anxiety. Telemetric monitoring of subjects’ heart rate (HR) and heart rate variability (HRV), which has been successfully used as a measure of autonomic regulation of cardiac activity to assess stress and well-being in companion animals over the last decade [32], was used to measure psychological changes and anxiety states in dog throughout the experimental sessions. Additionally, we tested the hypothetical link between contagious yawning and empathy. We hypothesized that if contagious yawning is related to dog’s capacity for empathy, then contagious yawning should follow the same familiarity bias as empathy, with dogs yawning more often at familiar than unfamiliar yawns.

## Materials and Methods

### Ethics Statement

The present study was conducted in strict accordance with the “Guidelines for the treatment of animals in behavioural research and teaching’’ by the Animal Behavior Society/Association for the Study of Animal Behaviour” and approved by the Ethics Committee of the Wildlife Research Center at Kyoto University (Japan) (No. WRC2010EC001). Dogs were recruited through owners’ responses to flyer postings at veterinary hospitals and kennels. Written informed consent for participation in this study was obtained from the owners.

### Data Collection

A total of twenty-five dogs older than 12 months of age served as subjects of the study (females = 13; males = 12; mean age: 5.9 years; Table 1). All dogs were companion dogs that lived in human households. Subjects were tested individually at the participants’ home, in rooms familiar to the dogs. Dogs were given a period of time to adapt to the new environment (e.g., cameras and tripods), the heart rate monitor (see below), and the experimenters before testing commenced. Subjects were considered to be comfortable if after the habituation period they were resting or passive, showing little interest in the experimenters or the experimental devices. Only dogs that were comfortable around strangers were included in the study.

**Table 1:** Age, breed, sex, and total number of yawns observed in the yawning and control conditions (familiar and unfamiliar conditions combined).

ID	Age	Breed	Number of yawns	
	(months)	(sex)	yawning condition	control condition
1	60	Standard poodle (F)	2	0
2	74	Standard poodle (M)	2	0
3	80	Labrador (M)	0	1
4	23	Golden retriever (M)	0	0
5	57	Maltese (M)	1	1
6	111	Papillon (M)	1	0
7	15	Golden retriever (M)	0	0
8	116	Golden retriever (M)	0	0
9	102	Labrador (M)	0	0
10	103	Mixed (F)	0	1
11	38	Miniature poodle (M)	0	0
12	105	Mixed Catalan sheepdog (F)	0	0
13	112	Pekingese (F)	0	0
14	100	Pit-bull (F)	0	0
15	40	Mixed (F)	0	0
16	124	Mixed (F)	0	0
17	48	Greyhound (F)	0	0
18	53	Mixed German shepherd (F)	6	0
19	54	Mixed (M)	1	1
20	83	Siberian husky (F)	2	0
21	50	Siberian husky (M)	5	0
22	74	Chihuahua (F)	1	1
23	132	Miniature poodle (F)	2	0
24	26	Mixed (F)	2	0
25	17	Mixed (M)	0	0

F: female, M: male.

The testing consisted of four experimental conditions (i.e., familiar-yawn, familiar-control, unfamiliar-yawn, unfamiliar-control), each of them lasting 5 min. The conditions were separated by a 3 min interval that also acted as rest period for the dogs. Familiar yawns and familiar control stimuli were performed by the dogs’ owner, while unfamiliar yawns and unfamiliar control stimuli were performed by one female researcher. Following the procedure of Joly-Mascheroni et al. [22], in the yawning condition the model (i.e., the owner or the researcher) sat in front of the dog and attracted its attention by calling the dog by its name. When the dog established eye contact with the model, the model acted a yawning movement with vocalization. The model repeated this sequence for 5 minutes. The owner (or the researcher when the owner was part of the test condition) sat behind the dog quietly. No feedback was given to any of the dog’s responses.

The exact same procedure was followed during the control condition, except that the model stretched and held his/her mouth open and closed it again without vocalizing, instead of yawning. We used open-mouth movement as a control stimulus because it has many of the facial movements of yawning but without the social information. Moreover, since most of the previous studies examining contagious yawning in dogs used the same control stimulus [22]–[24], [26] the results are more directly comparable. The order of testing conditions was counterbalanced between subjects. Two cameras were set up on tripods to record the dogs’ responses during the testing sessions. Prior to the start of the test, researchers advised owners on displays and tempo so that yawns and mouth movements were broadly consistent across models. The number of yawns elicited in each condition were recorded in real time by one researcher and then verified by subsequent video analysis. A subset of the videos was coded by an independent observer who was *naïve* to the conditions, with 100% agreement on the number of yawns.

Dogs’ heart rate (HR) and heart rate variability (HRV) responses to the different experimental conditions were monitored using a Polar RS800CX™ digital system for telemetric measurements. Polar® human heart rate monitors are frequently used in animal studies to measure HR and HRV and have been validated for this use in cows [33], pigs [34], horses [35], and dogs [36]. Polar® monitor devices have been recently used in dogs to investigate their heart rate responses in different emotional and potentially stressful situations, demonstrating that Polar® devices have enough sensibility to detect changes in dog cardiac activity under mild distress situations (e.g. [36]–[39]). The Polar RS800CXTM devise weighed less than 150 g and dogs showed no signs of distress during device application. The devise was fixed by an elastic strap to the dog’s chest and then switched on. Dogs were left a period of time to acclimatize to the devise and the strap. The R-R interval recording, as well as the time data, was sent automatically to the watch-computer placed on the dog’s back or collar. The recorded data were later read and processed by a host computer. Prior to the start of the experiment, the heart rate device was activated and synchronized with the video recording of the behaviors in order to have a perfect match of the behavioral and physiological data.

### Data Analysis

Generalized linear mixed models (GLMMs) with a binomial distribution and a logit link function were used to examine the effect of different variables on the presence/absence of yawn contagion. The dependent variable was a binomial term of whether the dog yawned or not, and the type of stimulus (i.e., yawn, control movements), familiarity level (i.e., familiar, unfamiliar), gender similarity between owner and experimenter (i.e. same, different), dog’s sex and age, and number of presented stimuli were entered as fixed term. The number of presented stimuli varied across individuals (range, 10–24) mainly due to individual differences in the time required to re-establish eye contact with the dog after each stimuli. Dogs’ identity was entered as random factor (nominal variable). To examine whether the frequency of elicited yawns was affected by several factors, linear mixed models (LMMs) were used. The dependent variable was the frequency of yawns corrected by the number of times the subject was exposed to the stimuli on each condition (i.e., number of stimuli performed by the model). Dogs’ identity was entered as random factor, and familiarity level, and dog’s sex and age were entered as fixed variables.

For the HRV analyses the frequency domain indices were calculated using Kubios Heart Rate Variability Analysis Software 2.0 for Windows [40]. The parasympathetic index (PNS) was computed as HF/total power and the sympathetic index (SNS) as LF/HF [32]. Linear mixed models (LMMs) were used to investigate the effects of different factors on dogs’ HR and HRV, with the identity of the dogs entered as a random variable, and the type of stimulus, familiarity level, and testing order as predictor variables.

We used restricted maximum likelihood methods for model estimation. A step-up strategy (i.e., fixed factors were added to the model sequentially) was used, and Akaike’s information criteria (AIC) values were used to select the best (most parsimonious) model. We present only the effects of variables present in the best models, except when none of the independent variables was found to significantly affect the dependent variable, in which case the effects of all independent variables are presented. Analyses were run on R version 2.8.1 using the lmer function included in the lme4 package [41].

## Results

Thirteen out of twenty-five dogs yawned during the experiment (Table 1). Overall, yawning occurred at an average of 1.0 (sd = 1.5) during the yawning condition and 0.2 (sd = 0.4) times during the control condition (familiar and unfamiliar conditions combined).

Via GLMMs we verified which variables affected the occurrence of contagious yawning. Type of stimulus, familiarity level, gender similarity, dog’s sex and age, and number of presented stimuli were entered as fixed term. The only factor remaining on the best model was type of stimulus (Table 2). The presence of yawn contagion was significantly higher when dogs observed the model acting a yawn than when dogs observed the open-mouth actions (*ß = *1.309, P = 0.025). Age and sex of the dogs were not among the variables remaining in the best model, which suggest that male and female dogs older than one year of age were affected by yawn contagion to a similar degree.

**Table 2:** Variables in the best GLMM explaining the occurrence of yawn contagion.

Variables	Variance	*ß*	SE	z	P	95% CI
**Fixed factors**						
Intercept		−2.328	0.505	−4.608	<0.001	
Type of stimulus (yawn *vs*. control)		1.309	0.586	2.232	0.025	0.15–2.45
**Random factors**						
Dog identity	0.33					

*ß*: Coefficient; SE: Standard error; CI: Confidence Interval.

**Figure 1:**
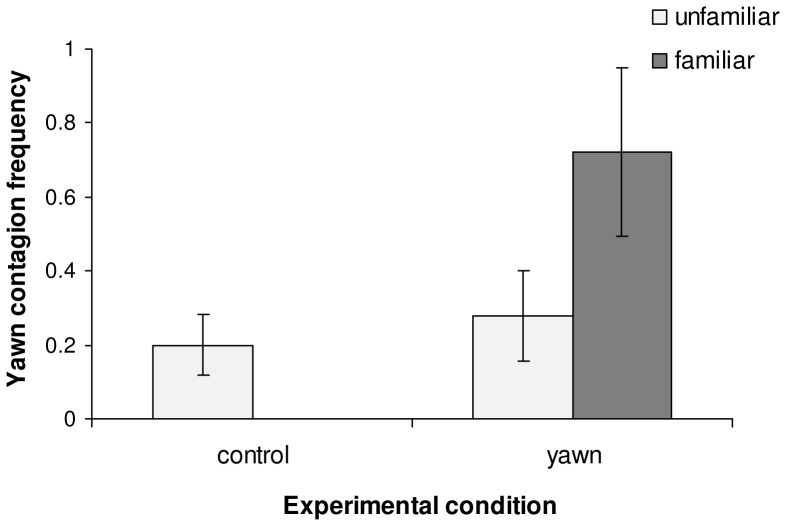
Yawn contagion in dogs as a function of the identity of the model (i.e. familiar *vs*. unfamiliar person). Bars represent mean (± SE) of yawn contagion frequency during yawning and control movement conditions according to the identity of the model.

To examine the factors that could explain the variation in the frequency of yawn contagion we used a LMM. Familiarity level and dog’s sex and age were entered as fixed variables. The only variable remaining in the best model was the identity of the model (familiar *vs*. unfamiliar model; *ß = *0.034, S.E. = 0.015, t = 2.197, 95% C.I. = 0.003, 0.06, P = 0.032; Figure 1). Observing a familiar individual yawning elicited in dogs more yawns than watching an unfamiliar human yawning.

Valid heart rate measures were obtained for 21 of the subjects. The HR monitor device could not be used in two dogs due to their small size. In two other dogs, there were several segments of missing data (or with artifacts) probably due to temporary poor electrode contact and/or movement of the dogs. These subjects were not included in the HR and HRV analyses.

**Figure 2:**
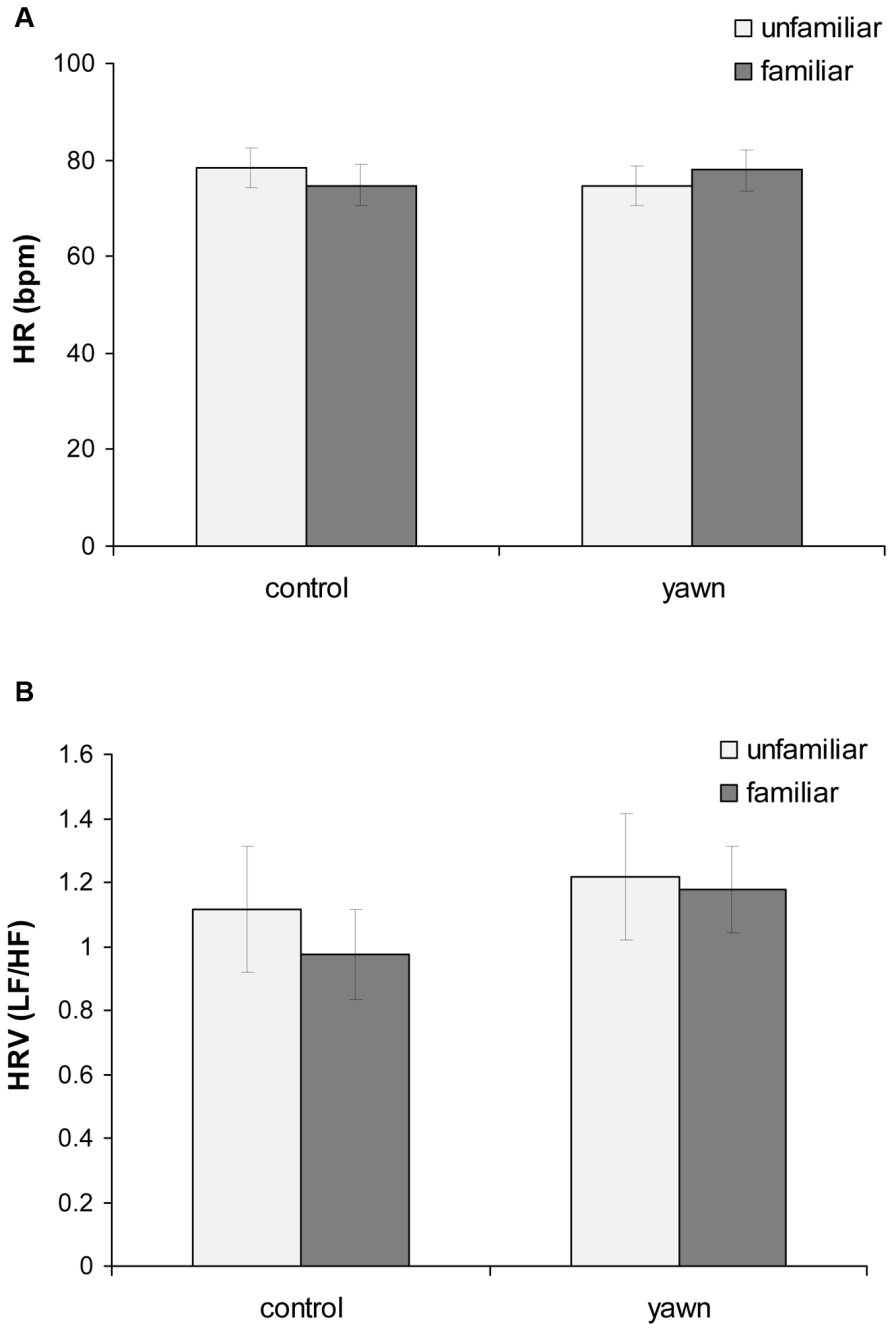
Heart rate (a) and heart rate variability (b) of dogs during the yawning and control movement conditions according to the identity of the model (i.e. familiar *vs*. unfamiliar person). HR: Heart rate; HRV: Heart rate variability.

Via LMMs we examined whether dogs’ HR and HRV were affected by different variables. Familiarity level, type of stimulus, and order of testing were entered as fixed factors. The results showed that none of the examined variables significantly affected dogs’ HR and HRV values (Figure 2). Dogs had overall similar HR and HRV across the entire trial session (testing order; HR: *ß* = −0.811, SE = 0.729, t = −1.11, P = 0.257, 95% C.I. = −3.53, 2.85; HRV: *ß* = −0.042, SE = 0.070, t = −0.601, P = 0.539, 95% C.I. = −0.179, 0.095). Furthermore, HR and HRV were not significantly higher when dogs observed a human yawning than when they observed control-mouth movements (HR: *ß* = −0.337, SE = 1.629, t = −0.207, P = 0.832, 95% C.I. = 70.01, 87.07; HRV: *ß = *0.149, SE = 0.156, t = 0.957, P = 0.329, 95% C.I. = −0.156, 0.456), neither were they affected by the level of familiarity (HR; *ß* = −0.018, SE = 1.630, t = 0.011, P = 0.991, 95% C.I. = −2.24, 0.61; HRV: *ß* = −0.087, SE = 0.156, t = −0.556, P = 0.570, 95% C.I. = −0.393, 0.219). Similar results were found when the analyses were limited to the subset of individuals that yawned contagiously during the yawn condition but did not yawn during the control condition (N = 8). Dogs’ HR and HRV were not significantly higher when subjects yawned in the yawn condition than when they observed control-mouth movements and did not yawn (HR: ß = 4.450, SE = 3.493, t = 1.274, P = 0.191, 95% C.I. = −2.39, 11.29; HRV: ß = 0.225, SE = 0.204, t = 1.099, P = 0.257, 95% C.I. = −0.175, 0.625).

## Discussion

The present study demonstrates that the presentation of human yawning is able to elicit yawns in domestic dogs and that the social bond, associated with empathy [10], mediates its occurrence. Interestingly, the physiological measures (i.e., HR and HRV) recorded continuously during the experimental sessions rule out the possibility that anxiety *per se* may have accounted for the observed pattern of yawning responses. Although this finding conflicts with previous studies on dog contagious yawning [23], [24], it corroborates the evidence reported by Joly-Mascheroni et al. [22], Silva et al. [25], and Madsen & Persson [26].

The discrepancy between results on dog contagious yawning is likely explained by the use of different methods of experimentation. For example, while studies using live models have been able to elicit contagious yawns in dogs [22], [26]; Harr et al. [23] and O’Hara & Reeve [24] failed to find such effect when they used video clips or a combination of both. Although some studies have successfully applied video or LCD playbacks to present stimuli to dogs [42], it is possible that videos are less ecologically relevant to dogs, and thus they attend differently to the videos and the live models. Another important methodological difference between studies is the type of sensory modality presented to the subjects. A yawn may include different sensory modalities (i.e. visual or auditory) and some studies have used only auditory cues (e.g. [25]), only visual (e.g. [23]) or a combination of both (e.g. [22], this study) with different results. However, before any conclusion could be drawn on which sensory modality elicited more contagious yawns in dogs; further investigations should explore the prevalence of each modality as well as the degree of individual variability to the sensibility to each.

It is also noteworthy that the expressions selected as control differ among studies, from silent mouth movements [22], [23], [26] to the sound of a yawn [24]. There is no consensus about what makes for the ideal control, and several facial expressions (e.g., smiles, silence mouth movements, species-specific expressions) seem to turn up baseline levels of yawning [28]. However, it has been documented that the mere sound of a yawn can be sufficient to elicit yawning in humans [8], [12], gelada baboons [16], and dogs: [25], and thus it seems unsuitable as a control stimulus. Furthermore, in the dog study using yawn sound as a control, the authors themselves stated that the “audio-only stimuli reported more yawn responses than any other condition” ([24], pp. 339), suggesting that a high proportion of dogs might have *actually* yawned contagiously during their study (11 out of 19 dogs yawned in response of the visual or auditory yawn stimuli but not at the mouth movements, from Table 3 in O’Hara and Reeve [24]). As Campbell and de Waal [28] suggested, further studies should focus on the impact of methodological variations on contagious yawning to facilitate comparisons across studies.

It could be argued that the silent mouth movements used as control stimuli in the present study could have the potential to impact our results. That is, the sound of a yawn could have drawn dogs' attention to a socially relevant stimulus (i.e. the mouth movements) during the yawning condition but not during the control one, since the control stimulus was silent. However, it has been reported that the perception of the eye region of yawning people is a potent stimulus in eliciting yawning, while yawning mouth is not [4]. Moreover, recent studies on children with autism spectrum disorder, who tend to spontaneously fixate more to the mouth than to the eyes when watching dynamic facial stimuli [43], repeatedly failed to show contagious yawning [12] except when they were instructed to fixate on the yawning eyes [44]. Thus, it seems unlikely that the possible more fixation to the mouth during the yawn condition increased dogs' probability to yawn contagiously.

On the other hand, it could be also argued that a combination of mouth movements and sound stimuli could have served as a releasing stimulus in the experimental condition but not in the control one, since the sound was not present. However, the empirical evidence from human and non-human animals shows that the presence of acoustic cues are not required to evoke a yawn since the mere view of a (silent) yawn is sufficient to elicit contagious (e.g. [11], [14], [15], [21]). Furthermore, using the same general design of the present experiment an additional group of dogs (N = 12, 7 females, 5 males, mean age = 51.9 months) were tested using open-mouth movements with vocalization as control stimuli (an “a” sound similar to the one produced during the yawning condition). Preliminary results show that while 33.3% of the dogs (N = 4) yawned during the yawning condition, none of the subjects did yawn during the control condition (McNemar Chi-square test: P = 0.045) suggesting that a combination of mouth movements and sounds *per se* does not work as a releasing stimuli for yawn contagion in dogs.

It has been suggested that contagious behaviors function to coordinate activities in group living animals [45], [46]. Therefore, it seems reasonable that the susceptibility to yawn contagiously is not specific to humans or primates but shared with other social species, since synchronizing behavioral activities has undoubted adaptive value for group-living animals. The contagious effect of human yawning on dogs may be interpreted in line with this argument: a communicative signal that helps to synchronize human-dog activities. Although there is anecdotal evidence that human yawns might produce similar, synchronous states in dogs [26], this hypothesis remains untested and further studies on the social function of contagious yawning in dogs are needed.

Most studies on yawn contagion in non-human animals have demonstrated the intra-specific effect of yawn contagion (chimpanzees: [14], [17], [19]; bonobos: [18]; gelada baboons: [16]; stumptailed macaques: [31]; budgerigars: [21]). However, studies on dogs have only been able to demonstrate cross-species (human-dog) contagious yawning. Dogs are unusually skilled at reading human social and communicative behaviors [47]. They can use human gaze and pointing to locate hiding food [27], [48], they respond to the attentional state of humans [49], and they can imitate human actions [50]. Thus, it is not surprising that they are also able to ‘catch’ human yawns.

However, it is puzzling that dogs have not responded in a similar way to the yawns of conspecifics. During domestication, dogs have become selected to maintain attention towards humans, which seems to be critical for dog-human communication and social learning [51]. Thus, it is possible that dogs are predisposed to respond more intensively, or only, to human social cues rather than conspecifics’ ones. However, observations of spontaneous social behavior of dogs [52]–[54], as well as experimental evidence on social cognition [55], do not support this hypothesis. Dogs use visual communicative signals, from body position to expressive use of eyes, lips, and teeth [52], and are able to use visual attention cues when interacting with other dogs [54]. Thus, it is also possible that the capacity for contagious yawning evolved as an adaptation for communication with conspecifics, and that this capacity was later transferred to dog-human interaction. The current experimental evidence, however, does not allow us to discriminate between these two possible explanations, since different methodology has been used to test intra and inter-species contagious yawning (i.e., videoed stimuli of conspecifics *vs*. human live demonstrators). The use of a standardized methodology in further investigations would be critical to understand dogs’ reactions to human and dog stimuli, which in turn will help us to gain insight into the evolutionary origin of contagious yawning.

An important implication of the present findings is that the contagion effect of human yawns in dogs is modulated by affective components of the behavior. Dogs yawned more frequently at the familiar yawns than at the unfamiliar, which is consistent with the observation that empathy is more pronounced the stronger the social attachment between individuals [10], [20]. Preston and de Waal [10] presented a theoretical model in which empathy is linked to all facilitation behaviors that rely on perception-action, including imitation and coordination, but also unconscious motor mimicry. According to this model, contagious yawning would be underscored by empathy and therefore individuals with a close emotional connection with the observer would be the most likely individuals to elicit contagious yawning [10], [11], [16], [17]. Through close cohabitation, dogs are able to establish close bonding and attachment with people. For instance, dogs show selective responsiveness to their owners and exhibit a range of attachment behaviors, i.e., search and proximity seeking behaviors, when separated from them [56]. Hence, the observed effect of familiarity on dogs’ contagious yawning probably reflects that positive affect may regulate unconscious motor mimicry in the domestic dog.

Some authors have suggested that familiarity bias would be also expected if an even lower-level mechanism underlies the phenomenon of contagious yawning [57]. According to this view, a yawn would be a special stimulus that “serves as a releaser to the unlearned behavior of others” [58]. In this scenario, a familiarity bias would be explained as a consequence of the different levels of attention of individuals toward different group members. That is, since subjects usually pay closer attention to close affiliates, attention bias rather than empathy differences would be responsible for the observed pattern. However, the studies that have controlled for levels of attention in animal studies do not support this view [17], [25]. Campbell & de Waal [17] and Silva et al. [25] measured the total amount of time subjects looked to the source of the stimuli (i.e. screen or speakers) finding that either there were no differences between familiar and unfamiliar conditions [25] or that subjects attended more to the unfamiliar yawns but yawned more to the familiar yawns [17]. Finally, in the present study a significant familiarity bias was also found after having controlled for the possibility to perceive the stimulus (i.e. the stimuli were presented only when the subject established eye contact with the model). Although these results cannot exclude the possibility that attention might have an effect on the responses of the subjects, they rule out the possibility that attention *per se* explains the observed pattern.

The importance of the social bond in shaping yawn contagion has also been demonstrated in humans [11], chimpanzees [17]; bonobos [18] and geladas baboons [16], with all studies reporting an association between the degree of bonding and the occurrence, rate, and/or latency of yawn contagion. The studies examining the empathic basis of contagious yawning in dogs have produced conflicting results, though. While two studies found an association between contagious yawning and empathy, with dogs yawning more at familiar than unfamiliar yawns [25]; two other studies failed to find such association [24], [26]. The problematic control stimuli used in O’Hara & Reeve’s study [24] (see above) raise questions about the interpretation of their negative results. On the other hand, Madsen & Persson [26] examined the ontogeny of contagious yawning, and their target sample was juvenile dogs (mean age = 7.23 months). Human and non-human primates show a developmental increase in susceptibility to yawn contagiously (humans: [6], [13]; chimpanzees: [14]; gelada baboons: [16]) which is suggested to reflect the developmental process of social cognitive skills, including the ability to identify other’s emotions [6]. Indeed, Madsen & Persson [26] found a similar developmental effect with only dogs above 7 months evidencing a contagion effect. Thus, it is possible that the social modulation of contagious yawning in dogs is more pronounced at older ages. In our study, only dogs older than 12 months of age were tested and a significant effect of the social attachment on contagious yawning was found. More data are clearly needed, and further studies could benefit from including a wider range of ages to clarify not only the empathic bias of contagious yawning, but also to better understand the social function of yawning in dogs.

Our findings go further in supporting the empathic bias of contagious yawning in dogs, since our methodological procedure allowed us to discard the alternative hypothesis that yawn responses were elicited by any kind of stressful event. In dogs, high frequencies of spontaneous yawns have been associated with middle tension states [30]. Thus, even if Silva et al. [25] found an effect of familiarity on contagious yawning (i.e., dogs yawned more frequently when they heard their owners yawning than when they heard an unfamiliar person yawning), their results could be interpreted in line with the tension hypothesis: hearing, but not seeing, their owners could produce uncertainty in the dogs and consequently evoke “tension yawns”. In our study, telemetric measures of dogs’ HR and HRV did not differ significantly between conditions, suggesting that the familiarity bias detected in this study was not due to changes in the subjects’ anxiety levels, but rather it reflects the modulating effect of the affective components of contagious yawning. Since the demonstration of the occurrence of contagious yawning in non-human species does not necessarily warrant that the underlying mechanism of the phenomenon is shared with human yawn contagion, further research should test for alternative hypothesis and control for factors that are known to affect the occurrence of yawning in animals.
